# Taking on SARS-CoV-2

**DOI:** 10.7554/eLife.80552

**Published:** 2022-06-28

**Authors:** Paola Kučan Brlić, Ilija Brizić

**Affiliations:** 1 Center for Proteomics, Faculty of Medicine, University of Rijeka Rijeka Croatia

**Keywords:** SARS-CoV-2, natural killer cells, innate immunity, ADCC, ADNKA, Viruses

## Abstract

A new study sheds light on how SARS-CoV-2 influences the way natural killer cells can recognize and kill infected cells.

**Related research article** Fielding CA, Sabberwal P, Williamson JC, Greenwood EJD, Crozier TWM, Zelek W, Seow J, Graham C, Huettner I, Edgeworth JD, Price DA, Morgan BP, Ladell K, Eberl M, Humphreys IR, Merrick B, Doores K, Wilson SJ, Lehner PJ, Wang ECY, Stanton RJ. 2022. SARS-CoV-2 host-shutoff impacts innate NK cell functions, but antibody-dependent NK activity is strongly activated through non-spike antibodies. *eLife* **11**:e74489. doi: 10.7554/eLife.74489.

Responding to a viral infection is a complex, multistep process that involves a multitude of immune actors. Innate immunity acts first, deploying a battery of cellular and molecular entities which are not specific to the invading pathogen. Natural killer cells, for instance, are powerful antiviral agents which can recognize and kill cells infected with a broad range of viruses (Björkström et al., 2022). An adaptive immune response is then mounted, which specifically targets the virus causing the infection. For example, antibodies precisely selected to bind to a range of viral proteins are produced and released in large numbers. In the case of SARS-CoV-2, the virus that causes COVID-19, both innate and adaptive responses are considered to be essential for the control of infection (Merad et al., 2022).

For natural killer cells to eliminate their targets, a number of stress-induced molecules must first be displayed on the surface of infected cells; there, they can be recognized by receptors on natural killer cells, a process which activates the cells’ killing programme. However, some natural killer cells also recognize infected cells by harnessing virus-specific antibodies produced by the adaptive immune response. This mechanism, known as antibody-dependent cellular cytotoxicity (ADCC), involves natural killer cells expressing an activating receptor which interacts with the tail end of antibodies.

Despite the efficiency of natural killer cells, viruses often have a broad arsenal of strategies at their disposal to escape these cells. Whether SARS-CoV-2 actively evades early natural killer cell response, and whether antibodies engage these cells via ADCC to protect against COVID-19, remains unclear. Now, in eLife, Richard Stanton and colleagues at various institutions in the United Kingdom – including Ceri Fielding of Cardiff University as first author – report results showing how SARS-CoV-2 interferes with the recognition processes of natural killer cells during the early stages of infection (Fielding et al., 2022).

First, the team screened which proteins are expressed on the surface of infected cells. This showed that SARS-CoV-2 actively evades natural killer cells by preventing the synthesis of several ligands that bind to natural killer cell’s receptors (Figure 1A). Further experiments revealed the identity of the SARS-CoV-2 proteins which could be responsible for this effect: the viral proteins Nsp1 and Nsp14, which could cooperate to reduce the expression of a number of surface proteins recognized by natural killer cells. The viral proteins likely perform this role by degrading the mRNA coding for the ligands and inhibiting translation in the cell; according to previous reports, this strategy has also been used against other factors involved in the innate immune response (Hsu et al., 2021; Thoms et al., 2020). Interestingly, however, recent evidence suggests that the related viral protein Nsp13 can actually increase the activation of natural killer cells by interfering with a receptor which inhibits the cells’ killing response (Hammer et al., 2022). How these opposing effects of SARS-CoV-2 affect the way natural killer cells control infections in vivo remains to be determined.

**Figure 1.**
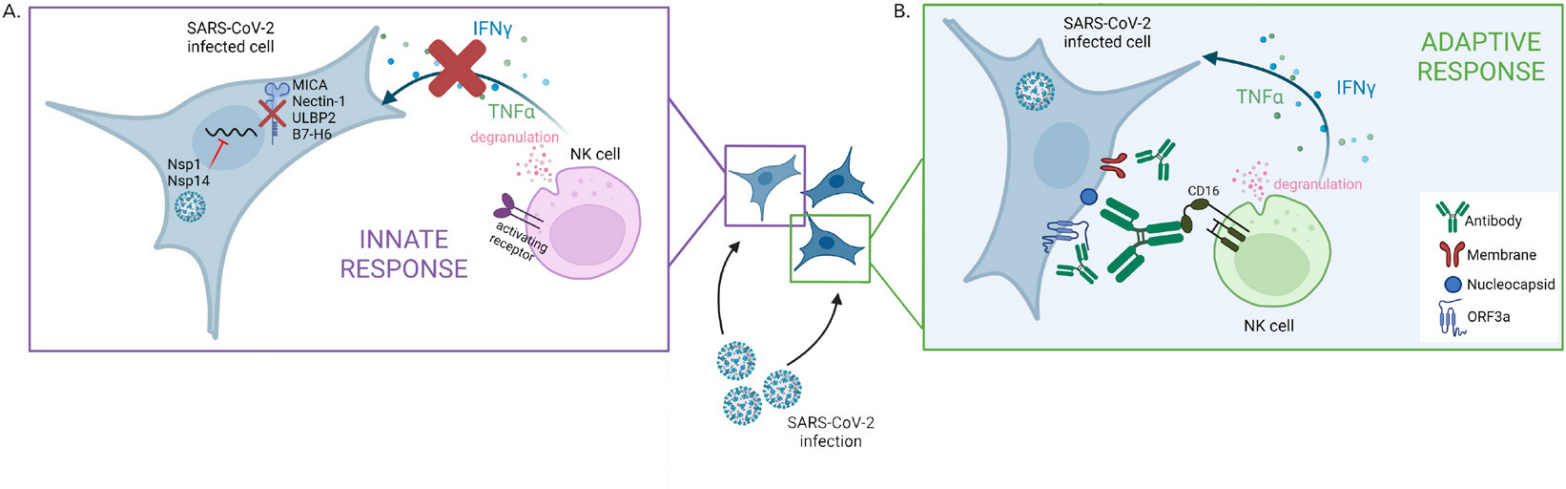
The two faces of the natural killer cell response against SARS-CoV-2. (**A**) SARS-CoV-2 infection interferes with the early activation of natural killer cells by having the viral proteins Nsp1 and Nsp14 prevent the synthesis of several surface proteins (MICA, Nectin1, ULBP2, B7–H6) that can activate natural killer cells. This leads to a dampening of that innate immune response, with impaired production of factors (IFNγ and TNFα) that promote the immune response and altered degranulation (a marker of the ability of natural killer cells to kill infected cells). (**B**) Antibodies produced against specific SARS-CoV-2 proteins – Nucleocapsid, Membrane and ORF3a – which are expressed on the membrane of infected cells, can efficiently trigger natural killer cell activation. This process takes place via CD16, an activating receptor on the surface of natural killer cells that interacts with the tail end portion of antibodies.

Figure created with Biorender.

Fielding et al. then showed that natural killer cells can be efficiently triggered by antibodies bound to SARS-CoV-2-infected cells (Figure 1B), demonstrating that the ADCC mechanism can activate these cells during COVID-19 infection. However, the antibodies triggering ADCC were not the ones targeting the spike protein, the viral component used in many current vaccines. In fact, further experiments revealed that vaccination-induced antibodies targeting the spike protein poorly engaged natural killer cells, a result in line with a study showing that vaccination-induced antibodies are not as good at mediating ADCC compared to infection-induced antibodies (Rieke et al., 2022). Fielding et al. then went on to reveal that the antibodies involved in ADCC were those produced in reaction to other viral proteins expressed at the surface of infected cells. In most COVID-19 patients, the infection-induced antibodies able to trigger ADCC persisted for at least six months.

Together, these results suggest that it could be possible to improve vaccine design by adding viral proteins which induce antibodies capable of triggering ADCC in natural killer cells to the current formulation. In addition, promoting natural killer cell activity by boosting ADCC response in patients with severe COVID-19 could become a therapeutic option, as these individuals show high levels of antibodies and impaired natural killer cell function (Merad et al., 2022; Witkowski et al., 2021).
